# New One-Pot Methodologies for the Modification or Synthesis of Alkaloid Scaffolds

**DOI:** 10.3390/md8082395

**Published:** 2010-08-24

**Authors:** Amir E. Wahba, Mark T. Hamann

**Affiliations:** 1 Department of Pharmacognosy, The School of Pharmacy, The University of Mississippi, University, MS 38677, USA; E-Mail: aewahba@olemiss.edu; 2 Department of Pharmacology, The School of Pharmacy, The University of Mississippi, University, MS 38677, USA; 3 National Center for Natural Products Research, The School of Pharmacy, The University of Mississippi, University, MS 38677, USA

**Keywords:** one-pot synthesis, cascade reactions, marine alkaloids, total synthesis, semisynthesis, organocatalysis

## Abstract

There are several avenues by which promising bioactive natural products can be produced in sufficient quantities to enable lead optimization and medicinal chemistry studies. The total synthesis of natural products is an important, but sometimes difficult, approach and requires the development of innovative synthetic methodologies to simplify the synthesis of complex molecules. Various classes of natural product alkaloids are both common and widely distributed in plants, bacteria, fungi, insects and marine organisms. This mini-review will discuss the scope, mechanistic insights and enantioselectivity aspects of selected examples of recently developed one-pot methods that have been published in 2009 for the synthesis of substituted piperidines, quinolizidines, pyrrolidines, hexahydropyrrolizines, octahydroindolizines and γ-lactams. In addition, progress on the synthesis of β-carboline (manzamine) alkaloids will also be discussed.

## 1. Introduction

The challenging task of developing a drug candidate from a natural source to clinical usage has many obstacles. Toxicity, development of resistance associated with the drug candidate, and providing a sufficient amount of material needed for preclinical and clinical studies, all represent major challenges. Although nature has been generous in providing structurally diverse molecules with varying biological activities, it is not always possible to obtain the required quantities of the desired drug candidate. This lack of material has inspired innovative chemistry that develops new methodologies, catalysts and inventive synthetic routes for the synthesis and modification of natural products.

Our research program has been involved in the isolation and the optimization process of a number of marine alkaloids and peptides. Among the most intriguing is manzamine A (**1**) with impressive antimalarial activity. A number of chemical stability challenges associated with its chemical modification require significant attention due to the complexity of the structure. This compound can be easily isolated from a marine sponge in good yields [1]. This inspired the development of new one-pot synthetic methodologies and conditions which are applicable to **1** and to other bioactive natural products.

During lead optimization of the manzamine alkaloids, the importance of one-pot methods for scaling certain drug leads became apparent. One-pot reactions are reactions in which two or more bond-forming transformations take place under the same reaction conditions without adding additional reagents or catalysts [2]. These reactions have numerous applications in the field of total synthesis of natural products, particularly in avoiding the costly protection/deprotection processes and purification of intermediates [2–6]. The importance of these reactions to the organic synthesis community is illustrated by the numerous reviews that have discussed different aspects of these reactions [2–11]. Most of these reviews discuss one-pot reactions that were developed during the total synthesis of selected examples of natural products (either as designed or serendipitous developments) such as Nicolaou’s [4–6] and Tietze’s reviews [2,3]. Although outstanding one-pot transformations designed for total synthesis of natural products were highlighted in these reviews, other one-pot transformations designed for other purposes were omitted. Synthesis of heterocycles by one-pot methodologies were discussed in detail in Padwa’s reviews [9,10]. Specific reviews on the construction of alkaloids are not found with the exception of Kobayashi’s review that discussed new methodologies developed for the total synthesis of some select examples of alkaloids [11].

Due to the growing interest in marine alkaloids, a detailed discussion of select, newly developed one-pot methodologies that have been utilized for the modification of marine alkaloids or the synthesis of common alkaloid moieties emphasizing asymmetric synthesis, are presented here. This mini-review discusses applications, mechanistic insights and enantioselectivity of recently developed one-pot methods that have been published during 2009. Those reports that could be applied in the synthesis of marine alkaloid scaffolds (*i.e.*, alkaloid moieties found in marine alkaloids) are emphasized. This mini-review will also cover our results for the manzamine alkaloids, as well as detailed asymmetric one-pot methods for the synthesis of substituted piperidines, quinolizidines, pyrrolidines, hexahydropyrrolizines, octahydroindolizines and γ-lactams.

## 2. New One-Pot Methods Used in the Modification of Marine Alkaloids

### One-pot reductive amidation of nitroarenes

**Figure 1:**
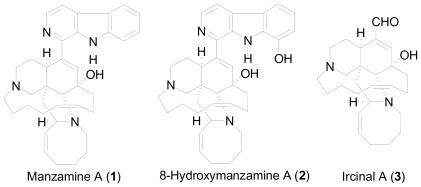
Manzamine alkaloids.

Manzamine alkaloids are a unique class of compounds that contain a complicated ring system coupled with a β-carboline moiety (Figure 1). The first representative of this family is manzamine A (**1**), which was first isolated by Higa and co-workers in 1986 [11]. This class of alkaloids has shown a wide range of biological activities (antimicrobial, antiparasitic, cytotoxicity, anti-inflammatory and pesticidal) and has also shown activity against HIV-1 and AIDS opportunistic infections [12]. The most potent activity for the manzamine alkaloids appears to be against malaria. Manzamine A (**1**) and its natural analog 8-hydroxymanzamine A (**2**) exhibited improved potency against malarial parasites both *in vitro* and *in vivo* relative to chloroquine and artemisinin [13]. However, the toxicity associated with high dose schedules limited the development of this class of compounds as new antimalarial drugs.

**Scheme 1:**
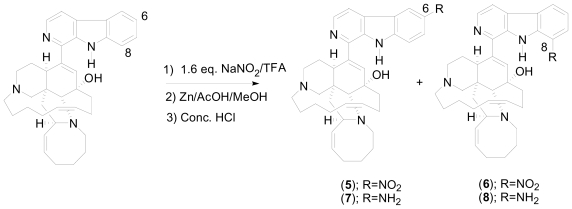
Nitration of manzamine A.

Since the mechanism of action of manzamine alkaloids as antimalarial agents is not completely clear; several structure activity relationship SAR and optimization studies using **1**, **2** or **3** as starting materials have been completed [14–18]. Nitration of **1** yielded 6 and 8 nitromanzamines and after reduction afforded the corresponding aminomanzamines (Scheme 1). Aminomanzamines are not stable, making them unsuitable for further amidation and *N*-alkylation reactions. However, 20 amide analogs were successfully synthesized in very low yield [15].

**Scheme 2:**
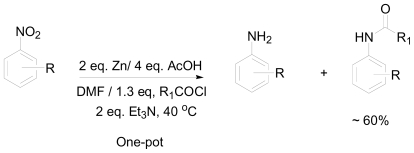
Optimized one-pot conditions for the reductive amidation of nitroarenes.

Inspired by the instability of aminomanzamines (**7** and **8**) and by the low yields of the amide derivatives, a one-pot reductive amidation method was developed for the conversion of nitroarenes to the corresponding amides [19]. The reduction of the nitro group generally requires a protic solvent as a carrier of the hydrogen generated *in situ*. However, the amidation reaction using acyl chloride requires aprotic solvents as well as dry conditions to prevent side reactions with the solvent. In addition, the amidation reaction using acyl chlorides requires basic conditions in which the base will neutralize the hydrochloric acid liberated from the reaction as a by-product. Because of this, a 3^o^ amine base (triethylamine, Et_3_N) was added in the reduction step where equimolecular amounts of acetic acid and zinc are used in addition to the acyl chloride. Once the amine is formed *in situ*, it immediately reacted with the acyl chloride facilitated by the tertiary amine present. Dimethylformamide DMF was shown to be the best choice of solvent for this reaction (Scheme 2). Using the optimized conditions, several nitroarenes were screened for one-pot conversion to amides. All the reagents were added at once and complete conversion of the starting materials to the corresponding amines and amides was observed with ~60% yield of the amides.

**Scheme 3:**
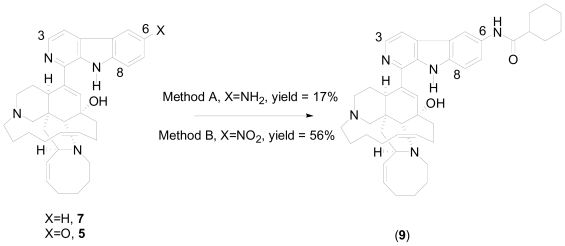
Synthesis of 6-cyclohexamidomanzamine A (**9**) by direct and reductive amidation approaches. Method A: 1.2 eq. cyclohexylcarbonyl chloride, 1.1 eq. Et_3_N, cat. DMAP, THF, rt, 1 h; Method B: 2 eq. Zn/4 eq. AcOH, 2 eq. Et_3_N, 1.2 eq. cyclohexylcarbonyl chloride, DMF, 40 °C, one-pot.

**Scheme 4:**
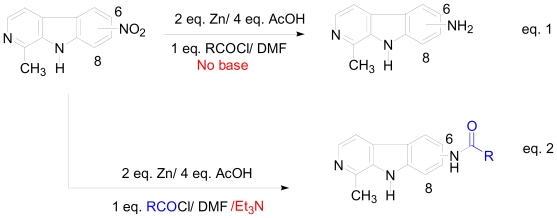
Validation of the built-in 3^o^ amine bases in manzamine A using nitro harmane as model compounds.

As a validation of this method, a one-pot approach was used for the synthesis of 6-cyclohexamidomanzamine A (**9**). This amide derivative showed potent antimalarial activity *in vitro* with an IC_50_ of 0.032 μM against the D6 clone of *Plasmodium falciparum*, and was synthesized from 6-aminomanzamine A (**7**) through the normal amidation pathway with low yield (17%)[15]. Starting with 6-nitromanzamine A (**5**), and using the optimized one-pot reductive amidation method, the yield of amide analog **9** was increased to 56% relative to 17% (Scheme 3). It was surprising that the reductive amidation of **5** proceeded without the addition of a 3^o^ amine base. A reasonable explanation for this observation is that **5** has two 3^o^ amine built-in bases which could possibly facilitate the reductive amidation reaction. To validate this explanation, harmane was nitrated to produce the closely related model compounds, 6- and 8- nitroharmanes. When the optimized one-pot reductive amidation conditions were applied to nitroharmanes without the addition of the Et_3_N, no amides were obtained, only aminoharmanes (Scheme 4, Equation 1); However, by adding Et_3_N the amide derivatives of harmane were isolated in good yield (60%)(Scheme 4, Equation 2). These experimental results validated the hypothesis regarding the built-in 3^o^ amine bases in manzamine alkaloids.

Although this method was developed particularly for the modification of manzamine alkaloids, it has clear utility in the synthesis of other alkaloids. The nitration of several biologically active natural products, as well as currently utilized drugs to generate the starting nitro materials for this one-pot reductive amidation method is ongoing. The goal is to show the general and practical applications for this method, as well as generating a library of compounds for further optimization and biological evaluation.

## 3. New One-Pot Reactions Used in the Asymmetric Construction of Some Important Alkaloid Moieties

### 3.1. One-pot asymmetric synthesis of substituted piperidines

**Figure 2:**
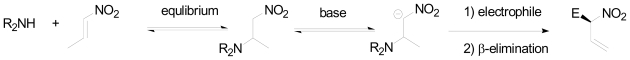
Intermolecular cross-double-Michael addition facilitated by amines via β-elimination.

Piperidine containing alkaloids are common in marine environments [20–22]. Although there have been several synthetic methods [23–25] reported for construction of this moiety, stereoselectivity remains a challenging task especially when three or more stereogenic centers or quaternary substituted carbons are present. The Shi group developed a novel cascade approach for the stereoselective synthesis of the piperidine moiety, based on their development of an intermolecular cross-double-Michael addition between α, β-unsaturated carbonyl compounds and nitroalkenes, facilitated by amines as Lewis bases (LB)[26]. Allylic nitro products are generated in the process via the β-elimination of the LB (Figure 2). The new cascade reaction encouraged Shi’s group to extend the cascade by involving an activated electrophilic intermediate for the construction of nitrogen containing heterocycles with two or more stereogenic centers.

**Figure 3:**
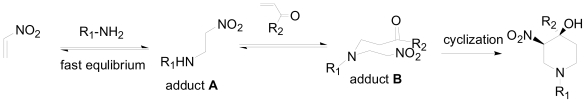
Proposed one-pot cascade piperidine synthesis.

**Scheme 5:**
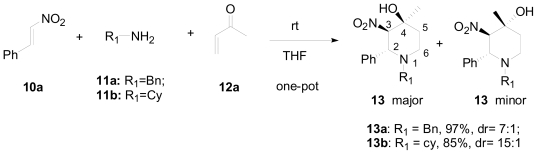
Optimized one-pot condensation for the asymmetric synthesis of piperidine moiety.

Based on their previous results, which showed that the addition of the amine to nitroalkene is fast, they proposed a one-pot cascade approach for the asymmetric synthesis of the piperidine moiety [27]. They postulated that adduct **A** (Figure 3), the addition product of the amine with nitroalkene, was suitable for Michael addition type reaction with an activated carbonyl to generate adduct **B**. Ring closure then provides the substituted piperidine motif in a one-pot approach with the generation of three stereogenic centers. To validate this proposed one-pot cascade sequence, nitrostyrene was allowed to react with methyl vinyl ketone (MVK) in the presence of a primary amine (Scheme 5). Being a complex mixture with many possible side reactions (*i.e.*, Baylis-Hillman reaction), it was surprising that substituted piperidines, **13a** and **13b**, were obtained in excellent yields and good diastereoselectivities with no side products. Solvent optimization of these one-pot conditions showed that THF gave the best yields with good diastereoselectivities (97%, dr = 7:1 for **13a**; 85%, dr = 15:1 for **13b**). The formation of **13** confirmed the formation of adduct **B** (Figure 3), which was trapped by a sequential Henry-aldol cyclization. Although three stereogenic centers were generated in piperidine **13** only two C-4 diastereoisomers were obtained, of which the *cis* isomer of C-3 nitro and C-4 hydroxy groups was the major product.

This one-pot reaction was compatible with a variety of nitroalkenes, amines and activated carbonyls. Different aryl and alkyl substituted nitroalkenes generated variation at the C-2 position. Moreover, both alkyl and aryl ketones were suitable for this cascade one-pot process giving different choices of substitutes on the C-4 position. Substituents on C-5 were possible through α-substituted enones, while β-substituted enones delivered variation at the C-6 position only when ammonia was used. It is interesting that only C-4 isomers were obtained in all cases.

**Scheme 6:**
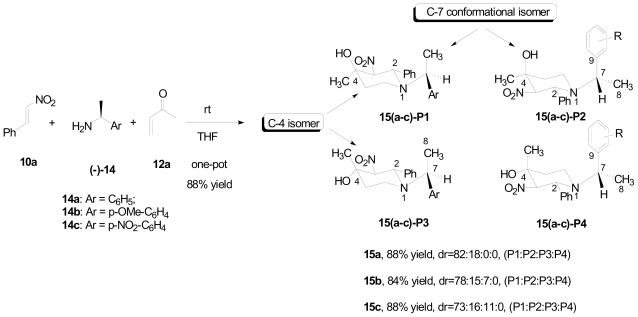
Chirality induction by chiral amines.

The aza-Michael adduct **B** (Figure 3) was not observed in reaction NMR studies; moreover, the piperidine products were stable under strong basic conditions. These results strongly suggested that the irreversible Henry-aldol cyclization was the rate determining step, which accounts for the piperidine diastereoselectivity through the chair transition state. With the formation of the C-2 stereogenic center during the amine conjugate addition, it is possible that the stereochemistry of the final piperidine product could be selectively induced by chiral amines through its involvement in the spatial arrangement of the chair transition state. In this case, a new stereogenic center on the exocyclic C-7 will be introduced on the piperidine product, which may result in the formation of four diastereoisomers. To investigate this proposed chirality induction, arylethanamines **14a**–**c** were applied in the one-pot piperidine synthesis and as expected, piperidines **15a**–**c** were obtained in good yields (Scheme 6). The results showed excellent diastereoselectivity of the C-4 position in **15a** and **15b** with dr > 10:1. In addition, modest chirality induction by the C-7 position was observed with dr = 4:1.

**Figure 4:**
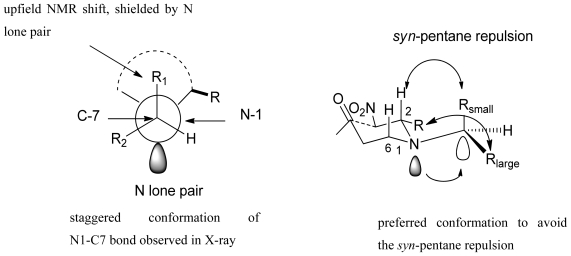
Proposed chair transition state and the exocyclic chirality induction.

X-ray analysis of the piperidine crystals revealed that the exocyclic C-7 adopted a staggered conformation with respect to the ring. Moreover, the chemical shifts of the C-8 and C-9 carbons were significantly shifted upfield in an anti-parallel position relative to the nitrogen lone pair electrons. This data suggest that the N1-C7 σ-bond rotation was restricted. Based on these results, a Henry-aldol cyclization chair transition state was proposed in Figure 4. The N-1 nitrogen lone pair electrons were placed in the axial position and the preferred staggered N1-C7 conformation could be achieved when the small group on C-7 was placed anti (axial orientation) to the N-1 nitrogen lone pair. Three *syn*-pentane interactions were expected in this staggered conformation (as shown in Figure 4); however, placement of the small group on C-7 anti to the lone pair will minimize these interactions. This proposed transition state was consistent with experimental observations. Also, the stability of this transition state could be influenced by the n-σ* electronic interaction between the nitrogen lone pair and the exocyclic axial small group.

**Scheme 7:**
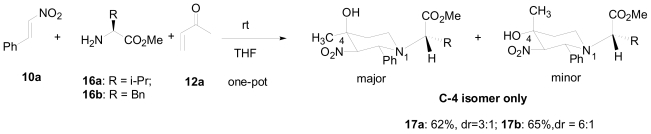
Enhanced exocyclic stereochemistry control by amino esters.

To validate the importance of this n-σ* electronic interaction in the chirality enhancement, the methyl group was replaced in the chiral amine with a carboxylate group which is less bulky and would produce a stronger n-σ* interaction. As expected, the reaction of the amino esters **16a**, **b** with enone **12a** and nitroarene **10a** yielded the corresponding piperidine products in moderate yields (Scheme 7). Interestingly, piperidines **17a**, **b** were obtained in their diastereomerically pure form. Complete chirality induction was achieved through control via exocyclic asymmetry. This new one-pot method opens the door for the asymmetric synthesis of several natural products that contain a piperidine moiety.

### 3.2. One-pot organo-catalytic synthesis of quinolizidine derivatives

**Figure 5:**
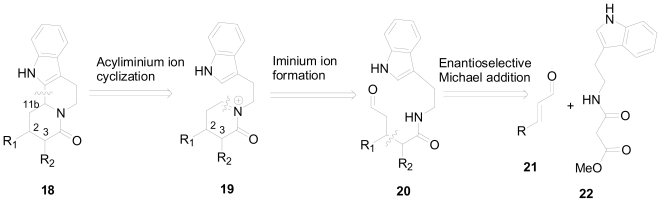
Retrosynthetic analysis of the indolo[2,3a]quinolizidine skeleton.

An elegant one-pot method was developed recently by Franzen and Fisher [28] for the asymmetric synthesis of substituted quinolizidines. This moiety is widely represented in several alkaloids isolated from plants, ants and marine organisms. Their retrosynthetic analysis for the indolo[2,3a]quinolizidine skeleton is highlighted in Figure 5. They postulated that the stereogenic center 11b could be generated through the asymmetric acyliminium ion cyclization of the imine (**19**). This imine could be obtained from the aldehyde precursor **20**, in which the aldehyde is apparently the adduct of an enantioselective Michael addition.

**Figure 6:**
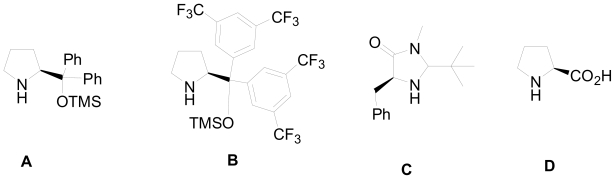
Organocatalysts used for the optimization of the one-pot conditions.

**Scheme 8:**
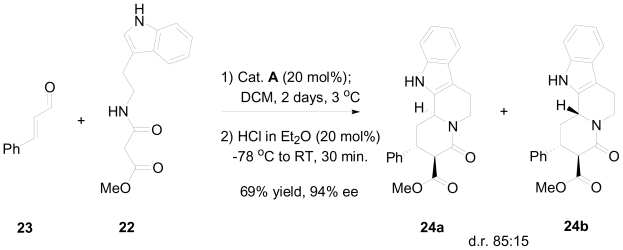
Optimized one-pot, two step conditions for the synthesis of indoloquinolizidine derivatives.

This retro-analysis required the addition of an activated amide **22** to the unsaturated aldehyde **21**, which has not yet been reported in the literature. To accomplish their synthetic plan, they optimized the enantioselective Michael addition of cinnamic aldehyde **23** and an activated indol substituted amide **22,** using different proline derivatives as organocatalysts (Figure 6), exploring different solvents, temperatures, as well as various acids needed for the cyclization step of the acyliminium ion (Scheme 8). Dichloromethane (DCM) was the solvent of choice and yielded compounds **24a** and **24b** with full conversion at room temperature, but with low enantioselectivity (88% ee). The enantioselectivity was increased to 94% by lowering the temperature to 3 °C, while further cooling (−20 °C) resulted in only 5% conversion. The proline derivative (*S*)-**A** showed the best selectivity, **B** was less selective and less active, while **C** and **D** were inactive for this reaction. When TFA was used in the acid-catalyzed cyclization of the acyliminium ion, the indoloquinolidine products were obtained nonselectively as a 1:1 mixture. However, some selectivity of **24a** over **24b** was observed when HCl was used. It was also noted that cooling down the reaction mixture prior to the addition of HCl increased the selectivity up to 85:15. The optimized conditions are highlighted in Scheme 8.

**Scheme 9:**
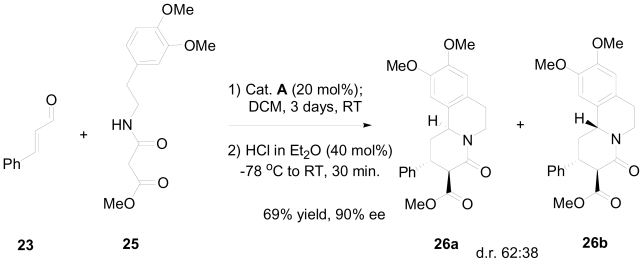
Optimized one-pot, two step synthesis of benzo[a]quinolizidine.

This one-pot, two step method was applied to several aromatic α, β-unsaturated aldehydes with good to excellent enantioselectivity. The indolyl moiety of **22** was replaced with an electron-rich phenyl group, which should give direct access to the benzo[a]quinolizidine skeleton. The reaction between **23** and the activated amide **25,** in the presence of the organocatalyst **A,** and subsequent addition of HCl, delivered the benzo[a]quinolizidines **26a** and **26b** with good to high enantioselectivity (Scheme 9). It was noted that the formation of the benzo[a]quinolizidine moiety required stronger acidic conditions (40 mol%) relative to the indolo[2,3a]quinolizidine (20 mol%). This is explained by the poorer nucleophilicity of the phenyl ring compared to the 3-indolyl moiety.

**Scheme 10:**
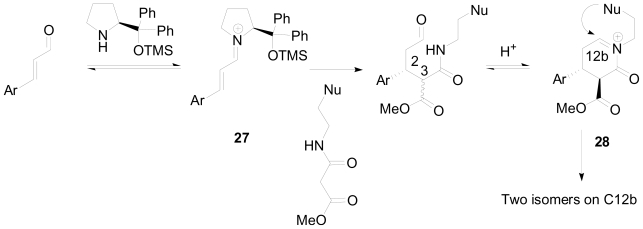
Proposed mechanism for the one-pot formation of quinolizidine moiety.

The absolute configuration of one of the benzo[a]quinolizidine derivatives was established by X-ray analysis as 2*R*,3*S*,11b*S*, which provided important mechanistic insight (Scheme 10). The aryl groups in the catalyst will shield the *Re* face of the iminium intermediate **27**, which will help to establish the *S* configuration on C-2 through the unshielded *Si* face. Intramolecular imine formation with epimerization of the stereochemically labile stereogenic center at C-3 will establish the more thermodynamically stable 2*R*,3*S*-*trans* configuration of the intermediate imine **28**. This acyliminium ion undergoes an electrophilic aromatic substitution with the aromatic moiety to give quinolizidine products.

**Scheme 11:**
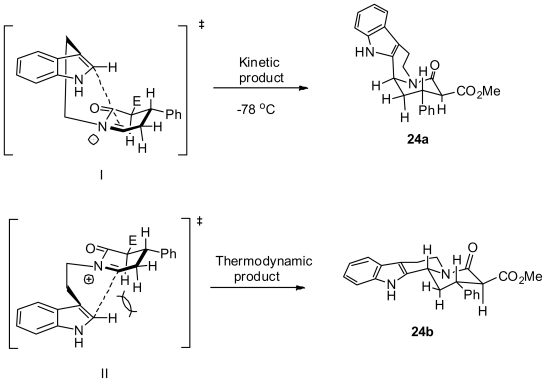
Kinetic *versus* thermodynamic product formation in the cyclization of the acyliminium ion.

The great diastereoselectivity observed in the acid catalyzed cyclization could be explained based on the reaction conditions. Considering the synthesis of indoloquinolizidines **24a** and **24b** (Scheme 11), the major isomer was **24a** with the indolyl moiety in an axial orientation. Although the formation of **24a** resembles a higher energy product, reaction via the transition state I (Scheme 11) is under kinetic control (−78 °C), owing to less steric hindrance from the equatorial α protons relative to the theromodynamic equatorial product.

**Figure 7:**
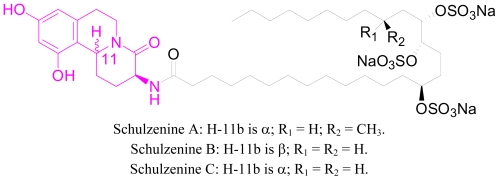
Schulzenine alkaloids.

**Figure 8:**
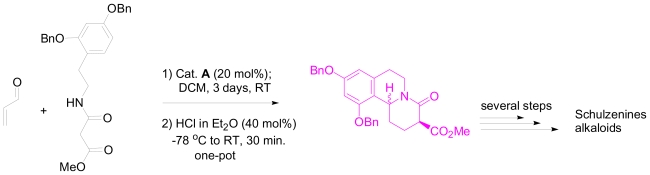
Proposed one-pot synthesis of the tricyclic core of Schulzenine alkaloids.

As a possible application of this one-pot method in the total synthesis of biologically active marine alkaloids, the tricyclic core of schulzenine alkaloids could be easily constructed using Franzen and Fisher’s method (Figure 7). Schulzenine alkaloids were recently isolated from the marine sponge *Penares schulzei* [29]. Schulzenine A–C inhibits α-glucosidase with IC_50_ values of 48–170 nM. Figure 8 represents the proposed one-pot reaction that could be used for the synthesis of the tricyclic core of schulzenine alkaloids.

### 3.3. One-pot enantioselective synthesis of pyrrolidine, hexahydropyrrolizine and related moieties

**Figure 9:**
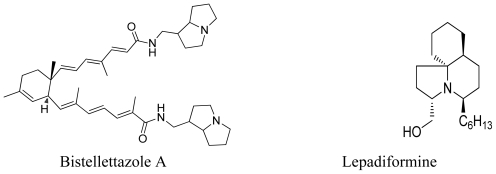
Marine alkaloids that possess hexahydropyrrolizine and octahydroindolizine moieties.

**Scheme 12:**
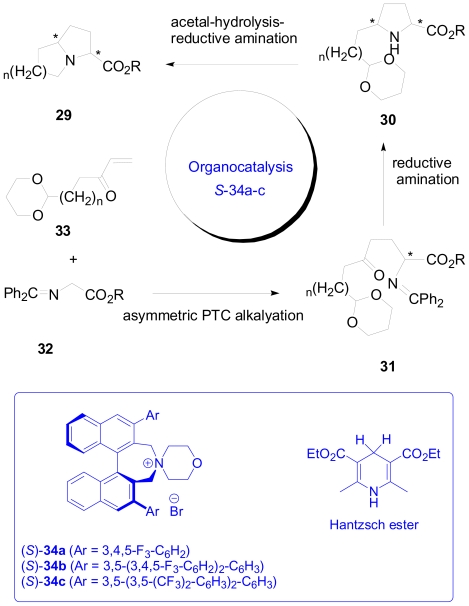
Organocatalytic retro-synthetic approach for the alkaloid core.

Recently, organocatalysis has been widely used in asymmetric synthesis due to its operational simplicity, low toxicity and ready availability compared to metal catalyzed reactions [30]. Maruoka and coworkers have used an organocatalyst of type (*S*)-**34** for one-pot construction of pyrrolidine, hexahydropyrrolizine and octahydroindolizine moieties with a high degree of stereoselectivity [31]. These core structures are commonly found in plant and marine alkaloids (Figure 9)[32,33]. Their organocatalytic based retro-synthetic analysis is presented in Scheme 12.

The key strategy is based on the asymmetric conjugate addition of the Schiff base of glycine ester **32** to α, β-unsaturated carbonyl compound **33** catalyzed by the chiral phase transfer organocatalyst (*S*)-**34a**–**c**. The organocatalytic Michael addition of **32** to **33** should deliver the adduct **31** in enantiomerically pure form. This adduct can then undergo intramolecular reductive amination facilitated by the Hantzsch ester to yield the pyrrolidine derivative **30** as an intermediate. Acetal hydrolysis followed by another intramolecular reductive amination of the intermediate **30** will furnish a bicyclic hexahydropyrrolizine skeleton.

**Scheme 13:**
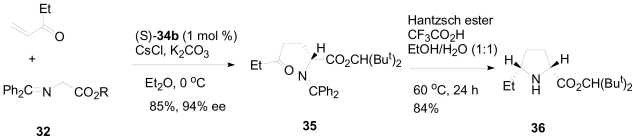
Optimized conditions for the conjugate addition and intramolecular amination steps in the synthesis of 2,5-disubstituted *cis*-pyrrolidine **36**.

First, the asymmetric addition of the glycine derivative **32** to methyl vinyl ketone (MVK), under the influence of an organocatalyst of type **34** (Scheme 13), was examined. Optimization of the reaction between **32** and MVK with K_2_CO_3_ and 10 mol% of CsCl at 0 °C gave the conjugate adduct **35**. This revealed that Et_2_O and the organocatalyst (*S*)-**34b** gave the best yield (85%), as well as the best diastereoselectivity (94% ee). Subsequently, optimization of the intramolecular reductive amination of **35** with the Hantzsch ester for the synthesis of the substituted pyrrolidine **36** was studied. The optimization process revealed that the Hantzsch ester (2 eq.) and trifloroacetic acid (1 eq.) in aqueous EtOH at 60 °C were the best conditions to build up the 2,5-disubstituted *cis*-pyrrolidine **36** stereospecifically in 84% yield.

**Scheme 14:**
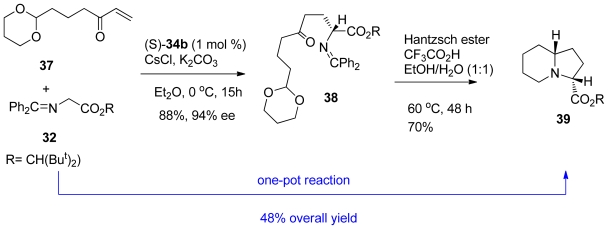
Synthesis of Octahydroindolizine **39**.

Next, the synthesis of the octahydroindolizine skeleton **39** using their optimized conditions (Scheme 14) was performed. Asymmetric conjugate addition of the glycine ester **32** to the enone **37** (2 eq.) and K_2_CO_3_ (5 eq.) under the influence of chiral phase transfer catalyst (*S*)-**34b** and CsCl in Et_2_O at 0 °C for 15 h gave the conjugate adduct **38** in 88% yield with 94% ee. Hantzsch ester mediated intramolecular reductive amination with subsequent acetal hydrolysis, followed by reductive amination, was effective to deliver the octahydroindolizine core **39** in 70% yield. The whole reaction sequence was done in a one-pot approach and performed without any difficulty by sequential addition of the reagents to afford **39** in 48% overall yield.

**Scheme 15:**
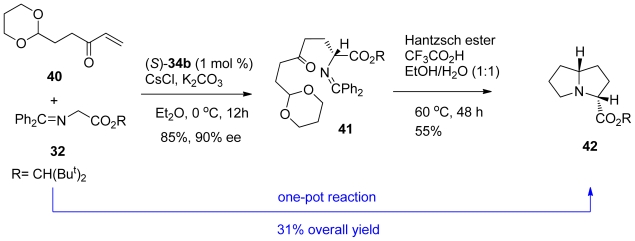
Synthesis of Hexahydropyrrolizine **42**.

Hexahydropyrrolizine **42** was also obtained by the same optimized conditions (Scheme 15). The asymmetric conjugate addition of **32** to the enone **40** using their optimized conditions yielded adduct **41** in 85% yield with 90% ee. It was noted that the enantioselectivity was slightly decreased by switching the cyclic acetal moiety of **38** to a 1,3-dioxolane (78% yield, 86% ee). The hexahydropyrrolizine skeleton **42** was then delivered in 55% yield by the action of Hantzsch ester mediated intramolecular reductive amination. The one-pot reaction was also possible with an overall yield of 31%.

### 3.4. One-pot diastereoselective synthesis of γ-lactams

**Scheme 16:**
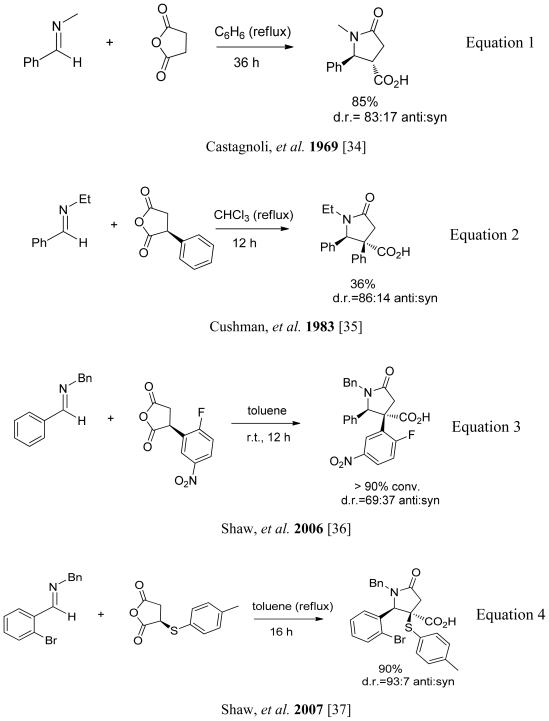
Milestones of the one-pot synthesis of γ-lactam moiety.

The cycloaddition of imines with succinic anhydride in a one-pot approach to construct the γ-lactam moiety in high yield was first reported three decades ago by Castagnoli *et al.* [34](Scheme 16, Equation 1). A mechanistic study was not published until 1983 when Cushman studied the electronic and steric effects on the stereochemical outcome [35]. A low yield was obtained when phenyl substituted succinic anhydride was used instead of succinic anhydride (Scheme 16, Equation 2). Also, variations in the structure of the succinic anhydride were not explored in detail in this methodology. Due to the wide abundance of the γ-lactam moiety in natural products and potential drug leads, the Shaw group has extensively studied the effect of substituents on the iminolysis of succinic anhydride derivatives. In their first report, Shaw and coworkers demonstrated the importance of electronic factors on the reactivity of phenylsuccinic anhydride when reacted with the imines derived from benzaldehyde and *o*-bromobenzaldehyde [36], demonstrating that the electron withdrawing groups in the phenyl ring give excellent yields (>90%)(Scheme 16, Equation 3). Following this yield improvement, Shaw and coworkers reported that sulfur-substituted succinic anhydrides also gave excellent yields (90%) and showed the highest diastereoselectivity (*anti* diastereoisomer) when reacted with different imines [37] (Scheme 16, Equation 4).

**Figure 10:**
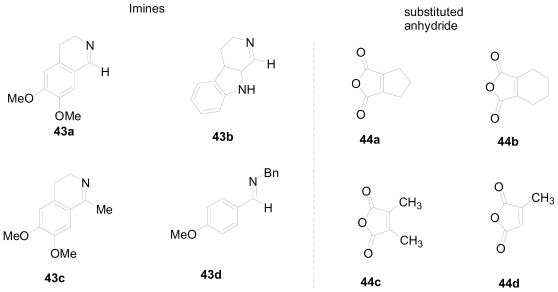
Imines and substituted maleic anhydrides used by Shaw *et al.* [38].

**Scheme 17:**
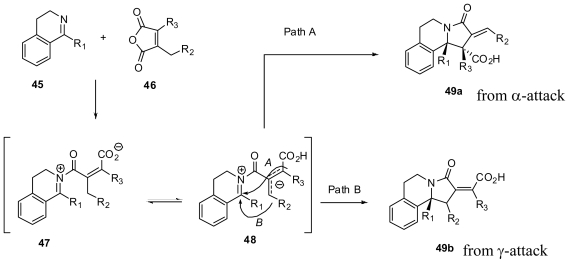
Possible cycloaddition pathways of imines with maleic anhydrides.

In the case of the iminolysis of maleic anhydride, a possible zwitterionic enolate intermediate could be generated through a prototropic shift which provides allylic stabilization. To validate this, Shaw and coworkers studied the cyclization of several polycyclic imines with maleic anhydride derivatives (Figure 10) as a new one-pot synthesis of complex nitrogen heterocycles [38]. Thus, the reaction of the imine **45** with maleic anhydride derivative **46** formed intermediate **47**, which could isomerize to **48**. Attack of the α- or γ-positions of the dienolate would lead to products **49a** or **49b** (Scheme 17).

**Scheme 18:**
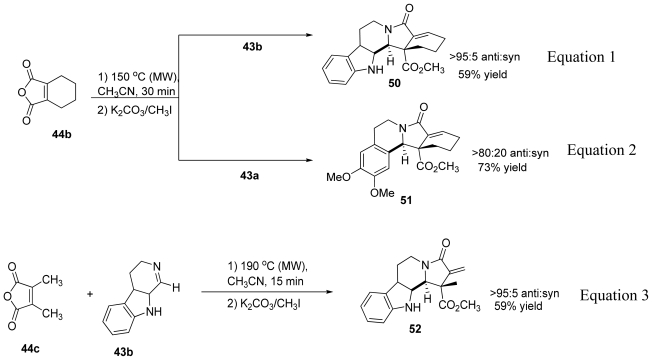
Polycyclic products from the reactions of anhydrides **44b** and **44c** with different imines.

Tetrahydrophthalic anhydride **44b** reacted with several imines through path A to give the formal cycloaddition products with high diastereoselectivity (*anti* configuration) and acceptable yields (Scheme 18, Equations 1 and 2). It was interesting that the diastereoselectivity of the reaction of **44b** with **43b** was reversed when triethylamine and triethylammonium hydrochloride were present in the reaction mixture. Methyl and dimethyl-substituted anhydrides did not react with several imines; however, an exomethylene-substituted lactam was obtained in good yield (59%) with excellent diastereoselectivity (>95:5 *anti:syn*) when imine **43b** was used (Scheme 18, Equation 3). Acyclic imines derived from aromatic aldehydes were less reactive relative to cyclic imines.

**Scheme 19:**
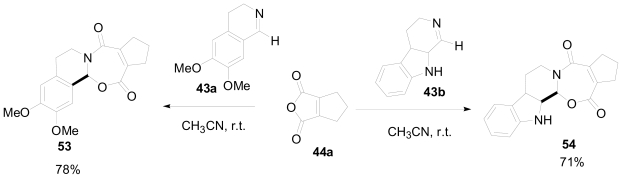
Reaction of anhydride **44a** with several imines to yield *N*,*O-*acetal products.

Although the ^1^H-NMR spectrum of the products obtained from the reaction of cyclopentane-fused maleic anhydride **44a** with several imines initially appeared to be consistent with the anticipated adduct, X-ray crystallographic analysis revealed the products to be *N*,*O*-acetals (Scheme 19). Mixing the anhydride **44a** with the imines **43a** and **43b** at room temperature yielded an immediate precipitate of *N*,*O*-acetals **53** and **54** in 78% and 71% yields respectively. No γ-lactam products were produced upon further heating.

**Scheme 20:**
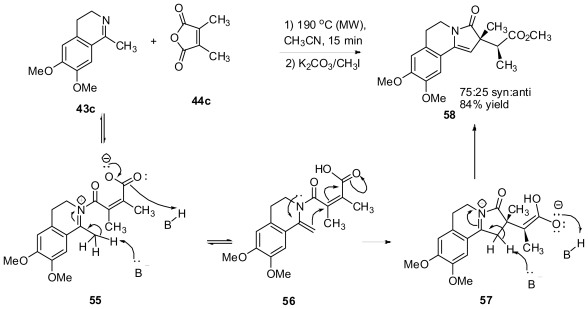
Mechanism of the formation of a new γ-lactam product obtained from the reaction of the ketimine **43c** with anhydride **44c**.

A completely different reaction manifold was observed in the reaction of ketimine **43c** with the maleic anhydride derivative **44c** (Scheme 20). A new γ-lactam product was obtained. The mechanism of formation of this new product is outlined in Scheme 20. A Prototropic shift of acylated imine **55** will yield the enamide carboxylic acid **56**, which is setup for an intramolecular Michael-type addition of the enamide to the unsaturated acid to deliver a new lactam **58** through enamide **57**.

**Scheme 21:**
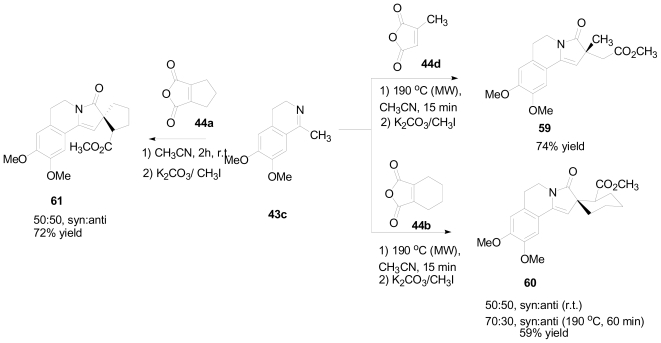
New γ-lactam products obtained from the reaction of the ketimine **43c** with several anhydrides.

Anhydrides **44d** and **44b** reacted similarly with ketimine **43c** to produce new γ-lactam products **59** and **60**, in yields of 74% and 59% respectively (Scheme 21). In the case of the spiro γ-lactam **60**, a 50:50 mixture of diastereoisomers was obtained after 15 min of reaction time. Continued heating of **60** under the reaction conditions shifted the diastereoselectivity to a 70:30 mixture, favoring the *anti* isomer. Product **61** was obtained in 72% yield when anhydride **44a** was reacted with ketimine **43c**. No stereoselectivity was observed in this case.

## 4. Conclusion

In this mini-review, we have highlighted a number of one-pot asymmetric methodologies developed for the synthesis of important alkaloid moieties. One-pot reactions are highly practical and ideal for scaling drug leads to gram scale and greater. The manzamine alkaloids are still providing inspiration for new chemistry in which one-pot methodologies are being developed and will be discussed in separate reports. We anticipate a greater application of these methods and similar methodologies in the field of total synthesis of marine alkaloids and have highlighted the possible route to the schulzenine alkaloids. Two or more methodologies could be combined to design a complete synthesis of a particular target. The alkaloid moieties highlighted in this mini-review (*i.e.*, substituted piperidines, quinolizidines, pyrrolidines, hexahydropyrrolizines, octahydroindolizines and γ-lactams) are common within the alkaloid family. Although there are many methods for the synthesis of these moieties, the development of one-pot methodologies to obtain high yields with a high degree of enantioselectivity will provide numerous opportunities for the synthetic community.
